# Low field magnetic resonance imaging of the equine distal interphalangeal joint: Comparison between weight-bearing and non-weight-bearing conditions

**DOI:** 10.1371/journal.pone.0211101

**Published:** 2019-01-28

**Authors:** Laurence Evrard, Fabrice Audigié, Lélia Bertoni, Sandrine Jacquet, Jean-Marie Denoix, Valeria Busoni

**Affiliations:** 1 Department of Clinical Sciences of Companion Animals and Equids, Equine Division, Diagnostic Imaging Section, University of Liège, Liège, Belgium; 2 Centre d’Imagerie et de Recherche sur les Affections Locomotrices Equines (CIRALE), Unité 957 BPLC, Ecole Nationale Vétérinaire d’Alfort, Normandie Equine Vallée, Goustranville, France; University of Pretoria, SOUTH AFRICA

## Abstract

This descriptive study aimed to compare the magnetic resonance appearance of the distal interphalangeal joint articular cartilage between standing weight-bearing and non-weight-bearing conditions. Ten forefeet of live horses were scanned in a standing low-field magnetic resonance system (0.27 T). After euthanasia for reasons unrelated to the study, the non-weight-bearing isolated feet were scanned in a vertical positioning reproducing limb orientation in live horses. The same acquisition settings as during the weight-bearing examination were used. Thickness and cross-sectional area of the distal interphalangeal articular cartilage and joint space were measured on tridimensional T1-weighted gradient echo high resolution frontal and sagittal images at predetermined landmarks in both conditions and were compared using a linear mixed-effects model. Frontal images were randomized and submitted to 9 blinded readers with 3 different experience levels for identification of weight-bearing versus non-weight-bearing acquisitions based on cartilage appearance. Weight-bearing limbs had significantly thinner distal interphalangeal cartilage (p = 0.0001) than non-weight-bearing limbs. This change was greater in the distal phalanx cartilage than that of the middle phalanx. Blinded readers correctly identified 83% (range 65 to 95%) of the images as weight-bearing or non-weight-bearing acquisitions, with significantly different results observed among the different readers (p < 0.001) and groups (p < 0.001). These results indicate that distal interphalangeal articular cartilage and particularly cartilage of the distal phalanx thins when weight-bearing compared to the non-weight-bearing standing postmortem conditions and suggest that cartilage abnormalities may be more difficult to identify on weight-bearing standing magnetic resonance imaging.

## Introduction

With the emergence of a low-field standing system, magnetic resonance (MR) imaging is increasingly used in the diagnosis of foot pain in horses. [1–4] Recent equine literature has addressed the possibility of evaluating articular cartilage using MR imaging, [5–11] and many MR studies have assessed articular cartilage on equine cadaver limbs. [5–8, 10–16] In recent publications on equine MR imaging, the articular cartilage thickness has been assessed on distal interphalangeal, [5] metacarpophalangeal [6, 10, 11, 17] and carpal joints. [12] Some studies demonstrated that MR imaging is reliable for cartilage thickness measurement when compared to histologic measurements of the carpal and metacarpophalangeal articular cartilage on isolated limbs when using high field magnets. [11, 12] However, other studies have failed to consistently correlate high field MR imaging measurement with gross cartilage thickness, particularly in the metacarpophalangeal joint. [17] Furthermore, some concerns have been raised about limitations associated with spatial resolution of the modality for assessing thin articular cartilage. [18] Moreover, whereas the limb is imaged in a non-weight-bearing position during high field MR imaging, it is weight-bearing during low field acquisition in a standing MR imaging unit, except in the few cases where the magnet is used in patients under general anaesthesia. The effect of loading on *in vivo* or *ex vivo* cartilage thickness has been well demonstrated in human orthopedics, e.g. in the knee [19–24], hip [25], and ankle, [26] and articular cartilage deformation is routinely assessed in human patients in the diagnosis of early degenerative joint disease. [20, 27–29] On the contrary, no report discusses the potential difference in cartilage appearance between weight-bearing and non-weight-bearing conditions in the equine patient. This study aimed to compare the appearance of the distal interphalangeal joint (DIPJ) articular cartilage between standing weight-bearing and non-weight-bearing conditions using a low-field MR system. We hypothesized that articular cartilage would have a different appearance in terms of thickness, delineation, homogeneity and signal intensity between standing weight-bearing and standing non-weight-bearing postmortem acquisitions. More particularly, we hypothesized that cartilage thickness would be less in images acquired in a weight-bearing position compared with non-weight-bearing limbs.

## Materials and methods

### Feet and MR images acquisition

This descriptive study was part of a larger research program approved by and conducted in accordance with French Institutional Animal Care and Use Committee. Ten forefeet of 5 non-lame live horses were scanned in a 0.27 T standing MR magnet (Hallmarq Veterinary Imaging Ltd, Guildford, Surrey, UK) using a dedicated hoof coil. Breed, age, sex and body weight from the horses are summarized in Table 1.

**Table 1:** Breed, age, sex and body weight of horses used in the study.

Horse	Breed	Age (years)	Sex	Body Weight (kg)
**1**	French Trotter	3	Mare	408
**2**	French Trotter	3	Gelding	454
**3**	French Trotter	4	Mare	468
**4**	French Trotter	5	Gelding	538
**5**	French Trotter	6	Mare	430
**Range**	-	3–6	-	408–538
**Mean**	-	4.2	-	459.6

A tridimensional high resolution T1-weighted (T1 3D HR) sequence (time of echo—TE: 8 ms, TR: 24 ms, slice thickness: 1.95 mm, matrix 512 x 512, pixel size 0.332 mm) was performed in the sagittal and frontal planes. Sagittal images were used to pilot the frontal images, in order to obtain a perpendicular orientation to the palmar portion of the glenoid cavity of the distal phalanx. The horses were euthanized for reasons unrelated to the study, both front limbs were transected at the level of the middle carpal joint and postmortem MR images were acquired within the 2 hours following euthanasia. The isolated feet were scanned in a vertical position reproducing limb orientation in live horses. The limbs were supported in position, with full solar contact to the ground, but were not loaded, so that the only load on the limbs was their own weight. The same MR sequences were acquired as during the ante-mortem weight-bearing study. Attention was paid during non-weight-bearing acquisitions to keep the foot positioning and frontal plane orientation as close as possible to the weight-bearing ones.

### MR imaging measurement and blinded reading

Articular cartilage was subjectively assessed on both image planes, by consensus between 2 readers (first and second authors, respectively ECVDI Diplomate and Associated Member). Homogeneity of signal intensity, delineation of the bone-cartilage and cartilage-synovium interfaces were evaluated.

**Fig 1:**
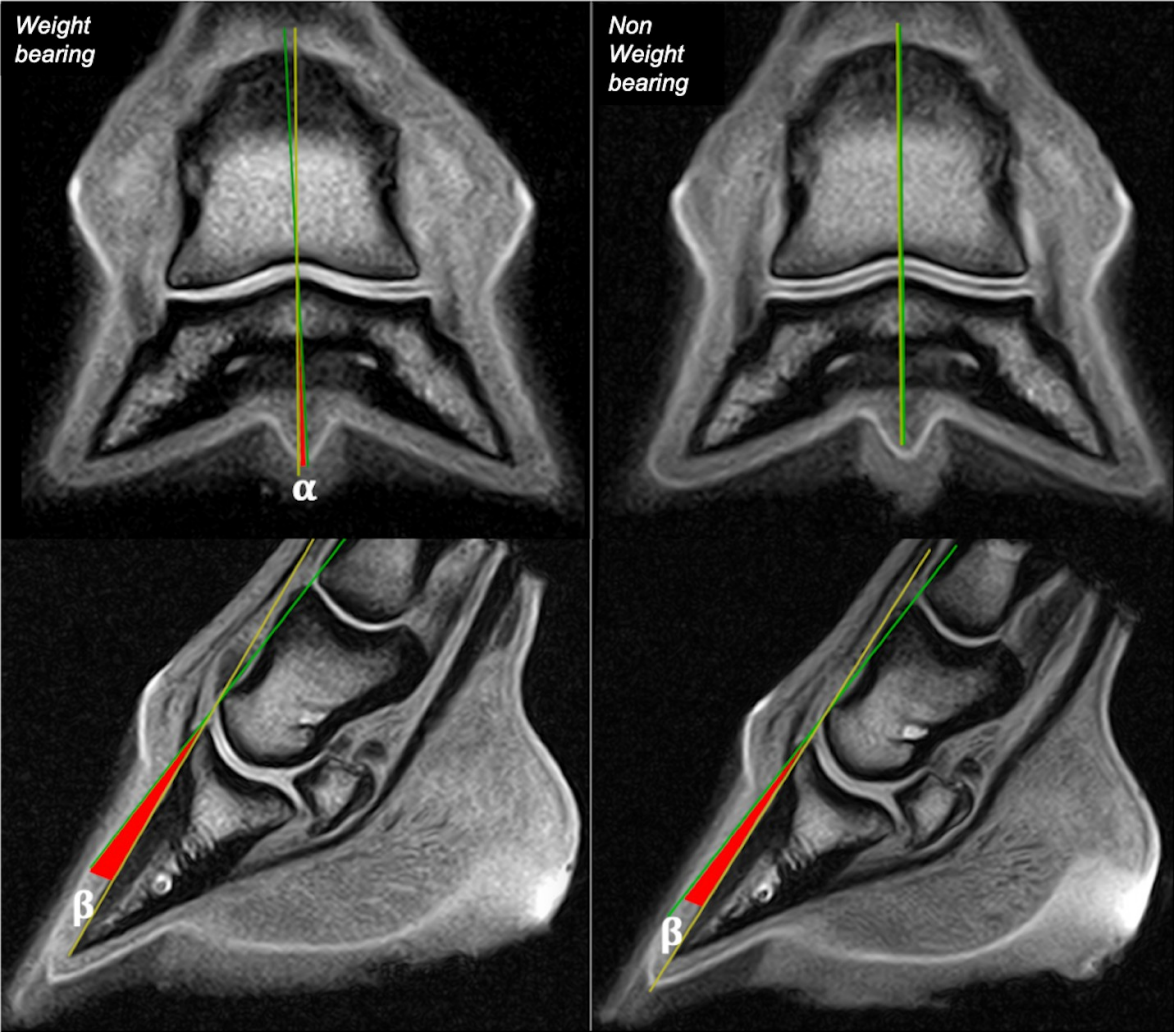
Illustration of lateromedial and dorsopalmar foot balance measurement. Upper images—Lateromedial balance: Frontal tridimensional high resolution T1-weighted (T1 3D HR) images from the same fore foot (left fore foot horse 3), obtained respectively during weight-bearing (on the left) and non-weight-bearing (on the right) acquisitions. Lateral is to the right. The angle α measured between the sagittal axes of the middle phalanx (green line) and of the distal phalanx (yellow line) represents the amount of varus or valgus deviation of the distal interphalangeal joint. Bottom images—Dorsopalmar balance: Sagittal tridimensional high resolution T1-weighted (T1 3D HR) images from the same fore foot (left fore foot horse 1), obtained respectively during weight-bearing (on the left) and non-weight-bearing (on the right) acquisitions. The angle β measured between tangential lines to the dorsal cortex of the middle (green line) and distal (yellow line) phalanges represents the amount of distal interphalangeal flexion.

Foot balance was evaluated as follow: two lines were traced in the sagittal plane of the middle and distal phalanx on frontal images. The distal angle α was measured between the 2 lines to evaluate lateromedial foot balance (Fig 1). Two lines were traced tangential to the dorsal cortices of the middle and the distal phalanx on sagittal images. The distal angle β between the 2 lines was measured to evaluate DIPJ flexion. (Fig 1).

**Fig 2:**
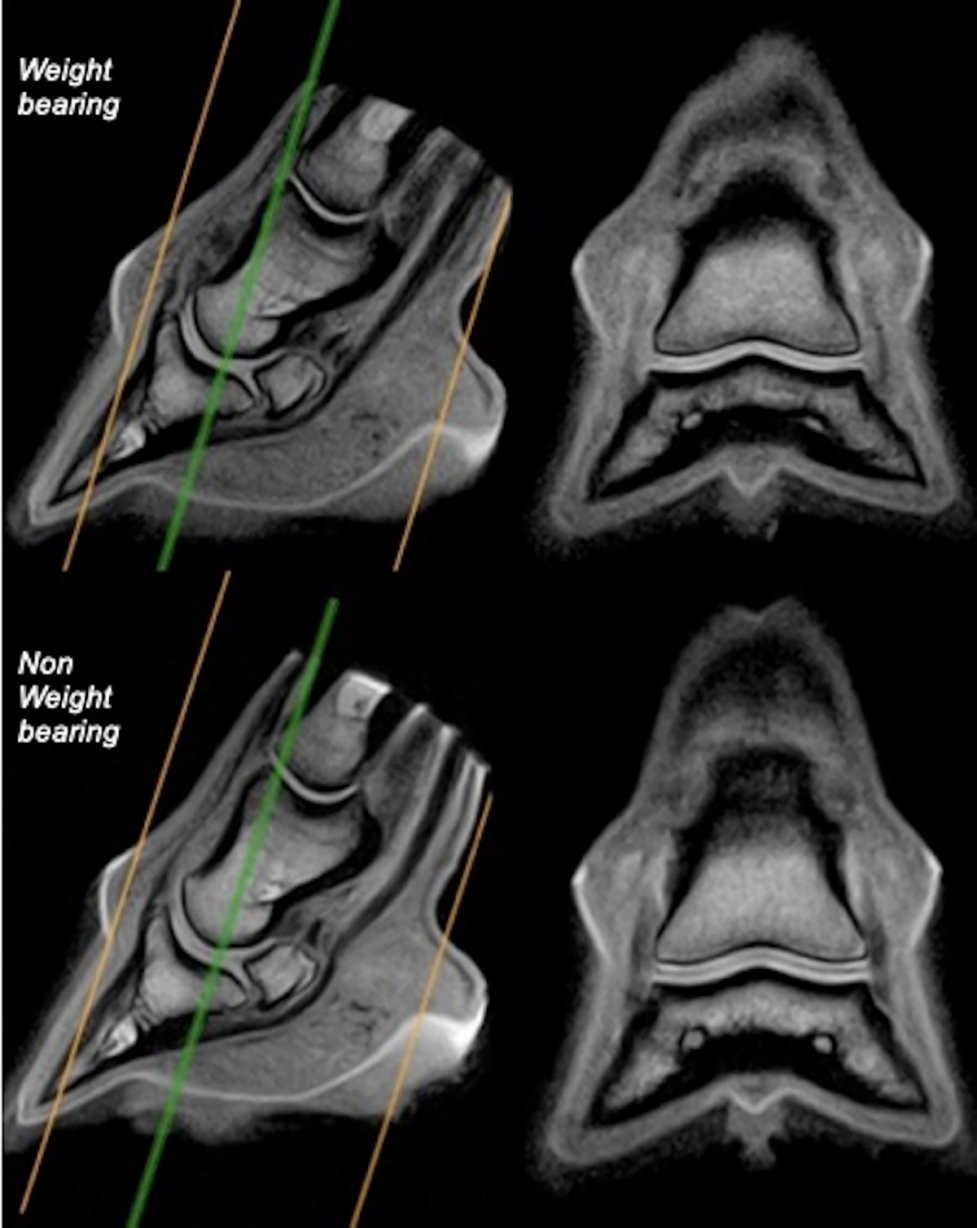
Illustration of frontal slice selection for articular cartilage measurements. Frontal tridimensional high resolution T1-weighted (T1 3D HR) images (on the right) are selected from sagittal T1 3D HR images (on the left) from the same fore foot, obtained respectively during weight-bearing and non-weight-bearing acquisitions. Lateral is to the left. Note perpendicular orientation of the frontal slice to the palmar aspect of the glenoid cavity of the distal phalanx. The orange lines represent the dorsal and palmar extremities of the field of view. The green line represents the selected frontal slice for measurements.

**Fig 3:**
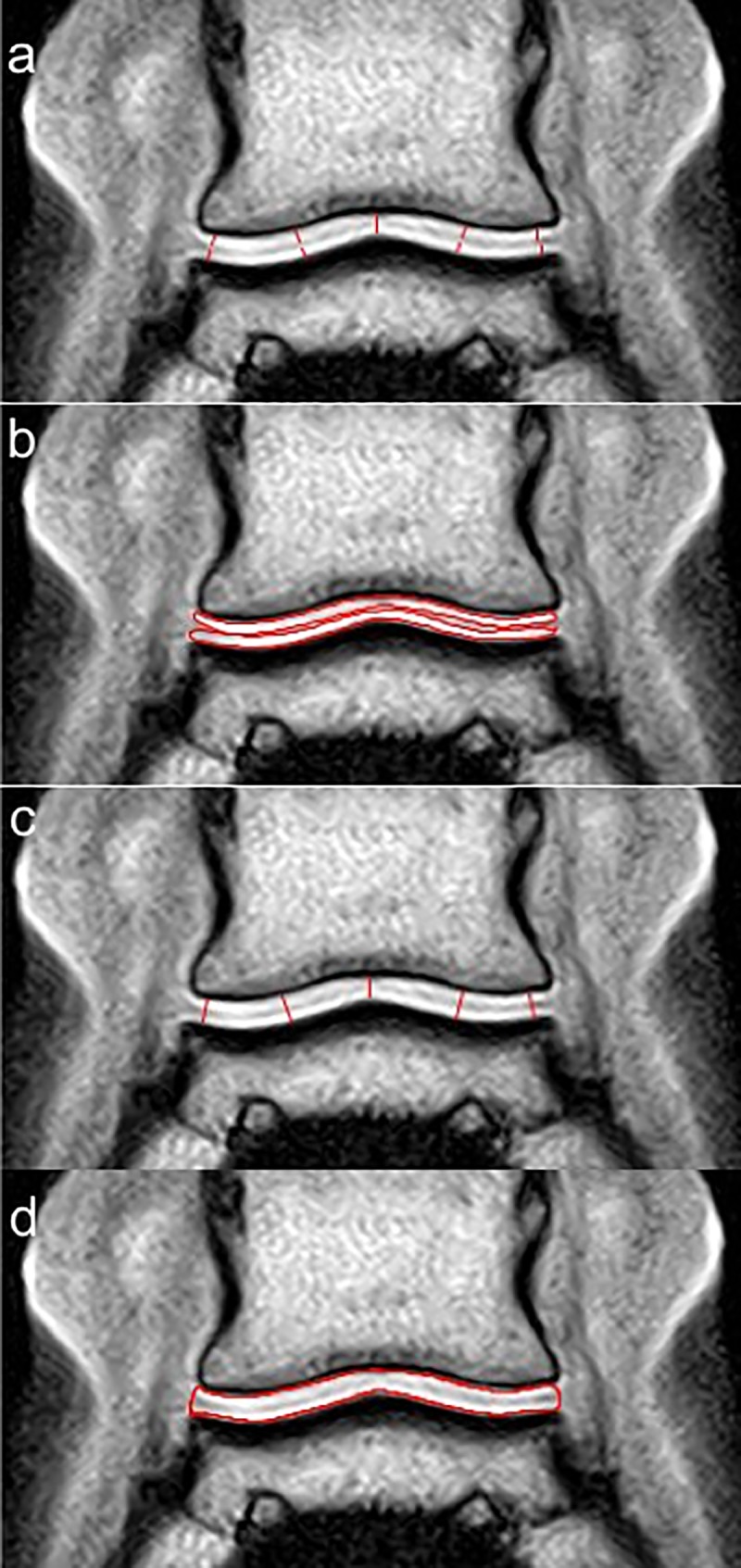
Illustration of articular cartilage thickness and area measurements on a frontal tridimensional high resolution T1-weighted (T1 3D HR) image of the distal interphalangeal joint of a forefoot. a. Individual measurements of articular cartilage thickness of the middle and distal phalanges, respectively, at 5 predetermined locations (abaxial and central aspects of medial and lateral condyles, and sagittal aspect). b. Individual measurements of articular cartilage areas from the middle and distal phalanges, respectively. c. Measurements of distal interphalangeal space thickness, combining articular cartilage from middle and distal phalanges (same locations as individual measurements in a). d. Measurement of distal interphalangeal space area, combining articular cartilage from middle and distal phalanges. Lateral is to the left.

**Fig 4:**
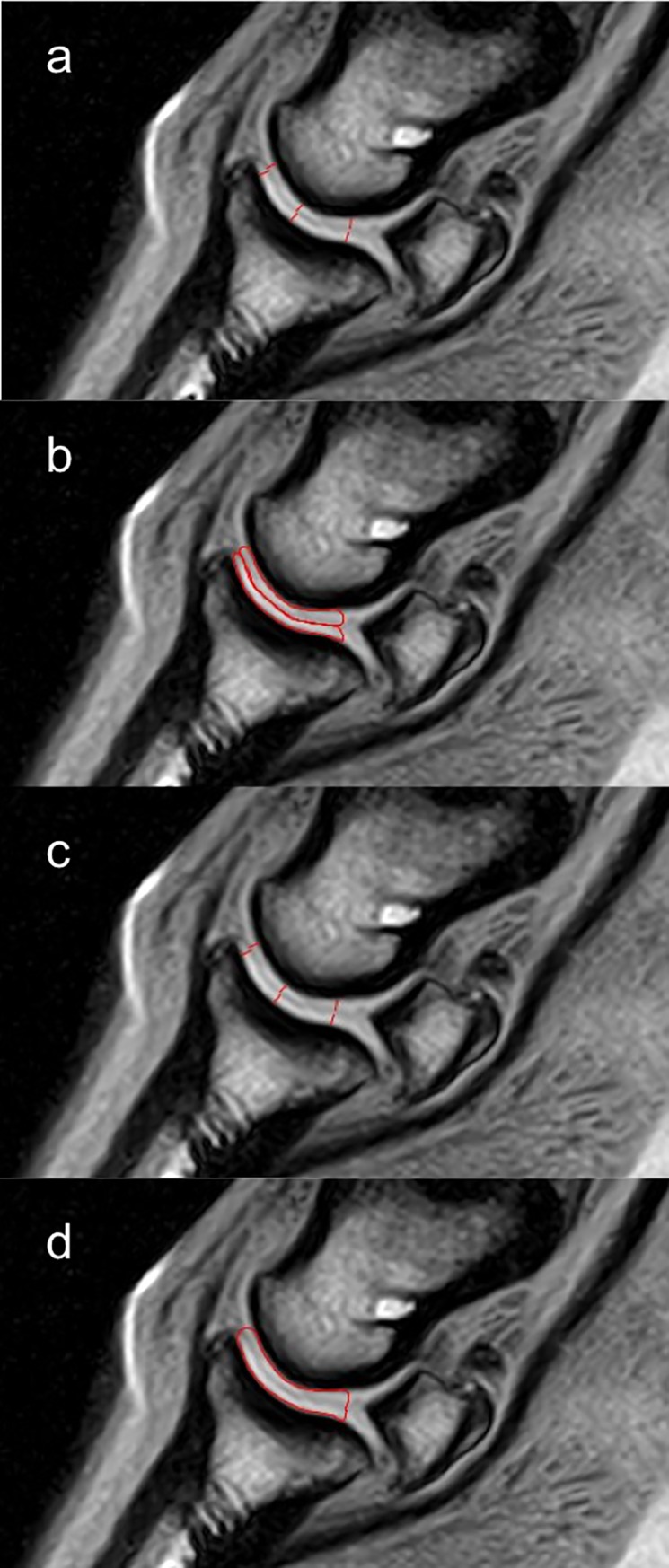
Illustration of articular cartilage thickness and area measurements on a sagittal tridimensional high resolution T1-weighted (T1 3D HR) image of the distal interphalangeal joint of a forefoot. a. Individual measurements of articular cartilage thickness of the middle and distal phalanges, respectively, at 3 predetermined locations (dorsal, central and palmar aspect). b. Individual measurements of articular cartilage areas from the middle and distal phalanges, respectively. c. Measurements of distal interphalangeal space thickness, combining cartilage from middle and distal phalanges (same locations as individual measurements in a). d. Measurement of distal interphalangeal space area, combining cartilage from middle and distal phalanges.

Two images were selected from the frontal series of each foot in the palmar aspect of the DIPJ. The images were selected where the section plane was most perpendicular to the articular surfaces of the palmar half of the condyle of the middle phalanx and of the palmar aspect of the glenoid cavity of the distal phalanx (Fig 2). Three images were selected from the sagittal series of 9 feet. One sagittal series was not available at the time of sagittal measurements and therefore comparative measurements of that foot (LF foot horse 4) were not performed in the sagittal plane. One image was selected in the median plane of the DIPJ and 2 in the center of each middle phalanx condyle. The following measurements were made on both frontal (Fig 3) and sagittal (Fig 4) images for both weight-bearing and non-weight-bearing conditions: 1) DIPJ space thickness was measured by drawing lines from the hypointense subchondral bone surface of the middle phalanx to the subchondral bone surface of the distal phalanx, therefore including articular cartilage from both middle and distal phalanges. Measurements were obtained perpendicular to the subchondral bone surfaces at 5 predetermined locations in frontal images: abaxial and central aspects of the lateral and medial condyles of the middle phalanx and glenoid cavity of the distal phalanx, and sagittal aspect of the joint. Measurements were obtained at 3 predetermined locations in sagittal images: at the dorsal, central and palmar third of the glenoid cavity of the distal phalanx, excluding the most palmar aspect of the DIPJ where the middle phalanx articulates with the distal sesamoid bone. 2) DIPJ cross-sectional area was measured by drawing spline curves along subchondral bone margins of the middle and distal phalanges and joining them at the most abaxial aspect of articular cartilage on the frontal images, and at the most dorsal and palmar aspect of the glenoid cavity of the distal phalanx on the sagittal images, including the articular cartilage of both the middle and distal phalanges. 3) A subgroup of 5 feet from 3 horses (one foot from horse 2, both feet from horses 4 and 5) was selected, based on better separation of synovium surfaces from the cartilage of the middle and distal phalanges on the frontal images. The respective thickness of the articular cartilage of the middle and distal phalanges was measured on these 5 feet, at similar locations to those previously used to obtain the joint space thickness measurements, by drawing lines from subchondral bone to synovial surface of the cartilage. Individual thickness measurement of the cartilage of the middle and distal phalanges was also performed on the sagittal images of 9 feet, at similar locations to those previously used to obtain the joint space thickness measurements. 4) The respective cross-sectional area of the cartilage of the middle and distal phalanges was also measured separately by drawing spline curves along their subchondral and synovial margins and joining them abaxially on the frontal images or at the dorsal and palmar aspects of the glenoid cavity of the distal phalanx on the sagittal images. Those measurements were performed by a single operator (LE) with a dedicated image processing program (Image J32, W. Rasband, Maryland, USA, 2009). Measurements at all predetermined locations were acquired sequentially and repeated 3 times in succession on each separate image. The images were not randomly analyzed and the reader was aware of the conditions of the study and previous measurements. Thickness values were averaged per location, as well as area values. The coefficient of variation was calculated in order to determine the homogeneity of the measurements. The error in linear distance measurement was also calculated as the ratio of the pixel size and the thickness measured. [18, 30]

A second part of the study was dedicated to blinded recognition of weight-bearing versus non-weight-bearing images of each foot, based on the difference in thickness and delineation of articular cartilage and joint space. The original frontal images, previously used for measurements, were paired (corresponding images in both conditions, total 20 pairs). Image pairs were then randomized and submitted to 9 blinded readers distributed in 3 groups of 3 readers each (group 1: equine orthopedic surgeons or large animal imaging residents, group 2: small animal imaging residents with little experience in equine imaging, group 3: veterinary students). They were asked to identify non- from weight-bearing images based on the clearly stated hypothesis that non-weight-bearing cartilage would be thicker and easier to delineate. A comparative pair (not included in the measurement and reading studies) was provided as a reference. The readers were not aware of the laterality of the provided images. Percentage of correct identification was recorded for each reader.

### Statistics

After validation of the normal distribution of the data and their residues, a mixed linear statistical model [31] (R version 3.1.3) was used to test the relationship between DIPJ cartilage thickness and area and several independent variables. The differences in thickness and area between weight-bearing and non-weight-bearing acquisitions were assessed, in both the frontal and sagittal acquisitions. The difference in thickness between medial, sagittal and lateral aspects and between dorsal, central and palmar aspects of the articular cartilage during the same acquisition were assessed, in both the weight-bearing and non-weight-bearing conditions. The differences in cartilage thickness and areas between the middle and distal phalanges was also tested, when available (5 feet for frontal measurements, 9 feet for sagittal measurements). Finally, the presence of interaction between selected independent variables was assessed (between the lateromedial or dorsopalmar location of the measurement and the weight-bearing condition of acquisition, between the phalanx and the condition of acquisition), in order to test the potential influence of measurement location on the differences in thickness observed. Results were considered significant at p < 0.05.

For the second part of the study (blind reading), a Pearson’s chi-squared test was used to test the dependency between individual reader and result and between readers group and result.

### Postmortem gross examination

Isolated limbs were finally dissected in order to evaluate the articular cartilage at gross postmortem examination.

## Results

### Subjective evaluation of DIPJ MR appearance

Articular cartilage of the middle and distal phalanges appeared as a homogeneous, clearly high T1 signal intensity layer covering the hypointense subchondral bone, in both non- and weight-bearing conditions. Despite the use of a high-resolution sequence, the trilaminar structure of the articular cartilage was not consistently visible. Synovial fluid with intermediate T1 signal intensity, was interposed between the cartilage surfaces of middle and distal phalanges in both conditions. Delineation at the cartilage-fluid interface varied depending on the amount of synovial fluid present. No MR imaging evidence of a cartilage defect or alteration of signal intensity consistent with a cartilage lesion was noted on any acquisition of any foot.

Despite hoof trimming, mild differences in foot balance were observed between the feet, impairing a perfect reproducibility of frontal plane selection among different feet. A difference in foot balance was also observed between both acquisitions on the same foot. Distal interphalangeal varus and subjective medial narrowing of the joint space was observed in 8 feet (all horses except horse 4) during the weight-bearing acquisition, with a maximum angle of 4.5° to the medial off the axial plane. This was no longer visible or reduced on the non-weight-bearing acquisition, with a maximum angle of 3° to the medial off the axial plane. One foot of horse 4 was balanced during the weight-bearing acquisition but showed varus (3.3° to the medial off the axial plane) and medial narrowing of the DIPJ space on the non-weight-bearing acquisition. The other foot of horse 4 showed mild valgus (1.7° to the lateral off the axial plane) during the weight-bearing acquisition and varus (2° to the medial off the axial plane) during the non-weight-bearing acquisition. Furthermore, minor to mild differences in the degree of DIPJ flexion were observed between both acquisitions of the same foot, with a maximum flexion angle of 10°, despite an attempt to exactly reproduce *in vivo* foot positioning during the postmortem non-weight-bearing acquisitions.

### MR imaging measurements and blinded reading

**Fig 5:**
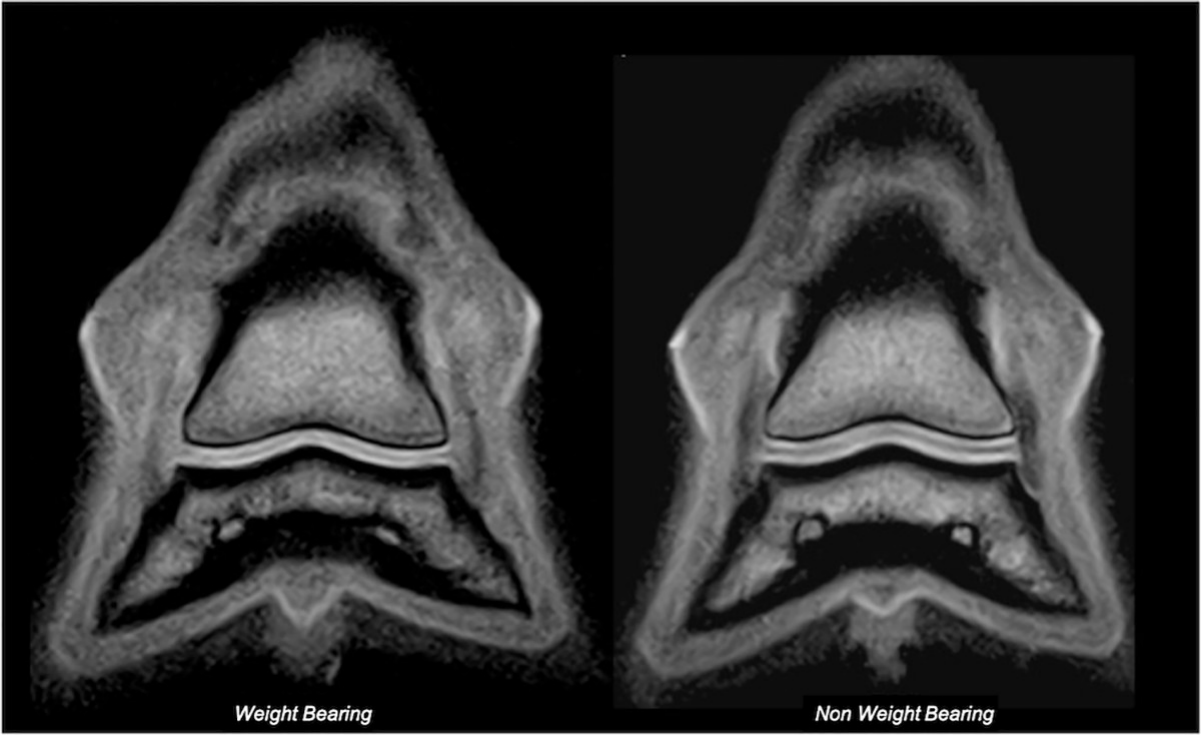
Comparison between tridimensional high resolution T1-weighted (T1 3D HR) frontal images from the same forefoot, respectively weight-bearing and non-weight-bearing, illustrating the difference in articular cartilage thickness and delineation between both conditions. Articular cartilage is thicker and better delineated on the non-weight-bearing compared to the weight-bearing images. Lateral is to the left.

**Fig 6:**
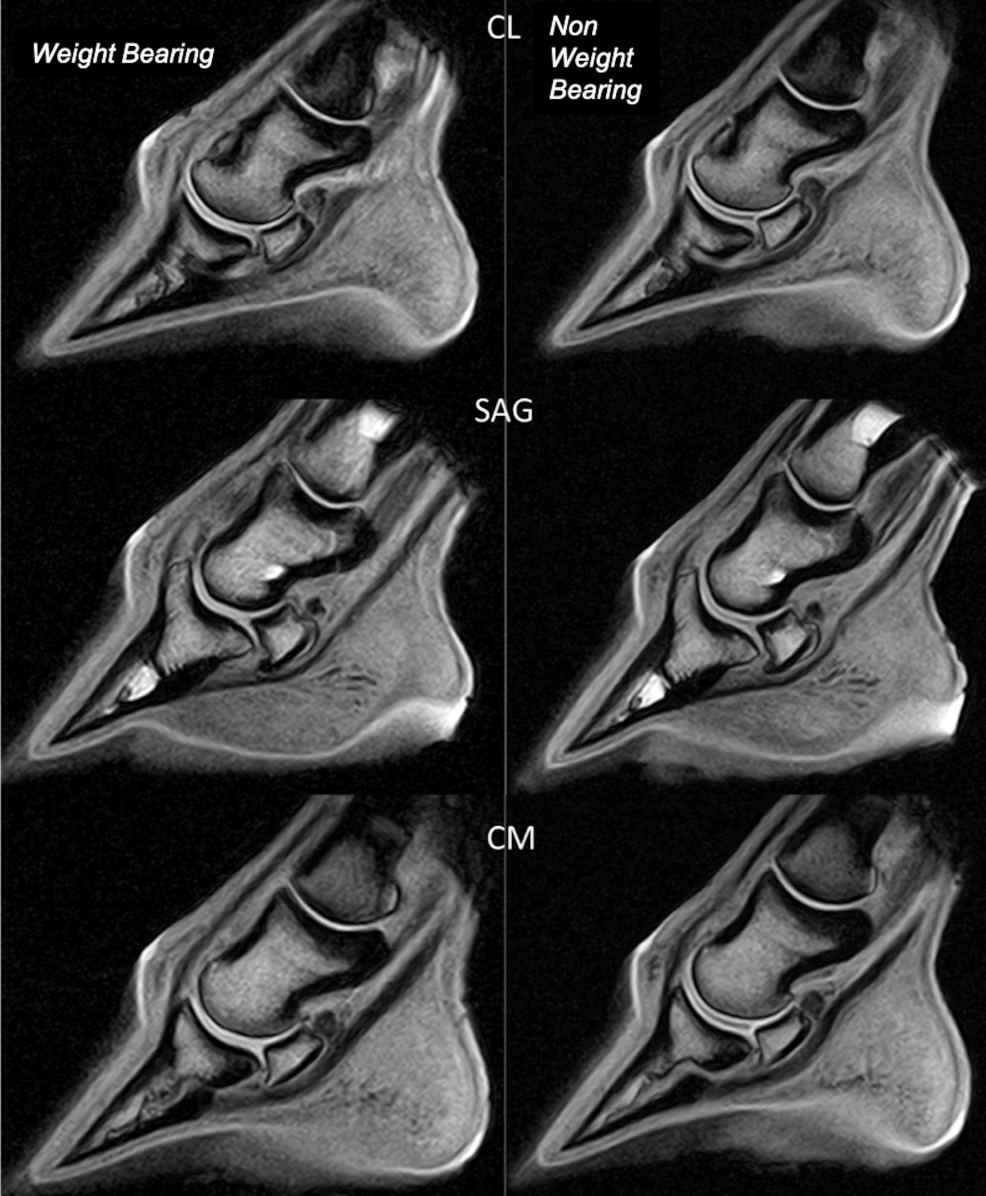
Comparison between tridimensional high resolution T1-weighted (T1 3D HR) sagittal images from the same forefoot, respectively weight-bearing and non-weight-bearing, illustrating the difference in articular cartilage thickness and delineation between both conditions. Articular cartilage is thicker and better delineated on the non-weight-bearing compared to the weight-bearing images. CL: centrolateral; sag: sagittal; CM: centromedial.

Averaged results for DIPJ space thickness, as well as individual cartilage thickness of the middle and distal phalanges and cross-sectional area measurements are summarized in Tables 2 and 3 and Figs 5 and 6. Complete datasets of frontal and sagittal measurements are available in S1 and S2 Tables. The mean cartilage measurements were significantly thinner and the areas significantly less in the weight-bearing DIPJ (p < 0.0001 and p = 0.0001) and distal phalanx (p < 0.0001) cartilage in both the sagittal and frontal planes, compared with non-weight-bearing cartilage measurements. Given a pixel size of 0.332 mm, percentages of error associated to the averaged linear thickness measurements ranged from 6% for DIPJ space to 39% for the individual cartilage of the distal phalanx (mean 18%) on the frontal images, and from 6% for DIPJ space to 83% for the individual cartilage of the distal phalanx (mean 21%). A coefficient of variation greater than 0.15 was found in 8/480 (2%) series of 3 measurements each and only one of those was greater than 0.2. A coefficient of variation greater than 0.15 was found in 52/648 (8%) series of 3 measurements each on the sagittal images.

**Table 2:** Mean values and standard deviation for distal interphalangeal joint space thickness and cross-sectional area (10 feet), articular cartilage thickness and area of middle and distal phalanges (5 feet) in weight-bearing and non-weight-bearing frontal acquisitions. P-values for Significant Effect of the Weight-Bearing Condition on the Cartilage Thickness and Area are included.

Thickness in mm									P-value	Area in mm^2^		P-value
	Weight-Bearing				Non-Weight-Bearing					Weight-Bearing	Non-Weight-Bearing	
	Med	Sag	Lat	Mean	Med	Sag	Lat	Mean				
**DIPJ**	3.06 +/- 0.87	3.35 +/- 0.60	3.85 +/- 0.49	3.43 +/- 0.77	4.04 +/- 0.57	4.14 +/- 0.72	4.37 +/- 0.53	4.19 +/- 0.60	<0.0001	172.86 +/- 28.86	208.54 +/- 45.52	<0.0001
**MP**	1.19 +/- 0.16	1.20 +/- 0.22	1.28 +/- 0.15	1.23 +/- 0.17	1.23 +/- 0.15	1.39 +/- 0.45	1.29 +/- 0.18	1.29 +/- 0.25	0.6223	59.21 +/- 8.57	63.68 +/- 11.77	0.6223
**DP**	1.14 +/- 0.22	1.05 +/- 0.21	1.07 +/- 0.12	1.09 +/- 0.18	1.53 +/- 0.43	1.58 +/- 0.34	1.61 +/- 0.47	1.57 +/- 0.42	<0.0001	51.25 +/- 8.85	76.44 +/- 20.42	<0.0001

mm: millimeters; mm^2^: millimeters square; DIPJ: distal interphalangeal joint; MP: middle phalanx; DP: distal phalanx; Med: mean medial values, averaged from the centromedial and medial abaxial measurements; Lat: mean lateral values, averaged from the centrolateral and lateral abaxial measurements; Sag: mean sagittal values. P-values represent the probability of a significant effect of weight-bearing condition on the measured cartilage thickness and area.

**Table 3:** Mean values and standard deviation for distal interphalangeal joint space thickness and cross-sectional area, articular cartilage thickness and area of middle and distal phalanges (9 feet) in weight-bearing and non-weight-bearing sagittal acquisitions. P-values for Significant Effect of the Weight-Bearing Condition on the Cartilage Thickness and Area are included.

Thickness in mm									P-value	Area in mm^2^		P-value
	Weight-bearing				Non-Weight-Bearing					Weight-Bearing	Non-Weight-Bearing	
	Dors	Centr	Palm	Mean	Dors	Centr	Palm	Mean				
**DIPJ**	2.90 +/- 0.49	3.22 +/- 0.47	3.37 +/- 0.45	3.15 +/- 0.51	3.08 +/- 0.61	3.83 +/- 0.66	4.20 +/- 0.57	3.69 +/- 0.77	<0.0001	73.97 +/- 13.22	86.48 +/- 17.35	<0.0001
**MP**	1.17 +/- 0.18	1.28 +/- 0.25	1.36 +/- 0.20	1.26 +/- 0.23	1.21 +/- 0.23	1.27 +/- 0.20	1.53 +/- 0.33	1.34 +/- 0.29	0.2515	29.17 +/- 5.84	29.59 +/- 6.37	0.2515
**DP**	1.06 +/- 0.26	1.25 +/- 0.33	1.66 +/- 0.18	1.19 +/- 0.28	1.05 +/- 0.21	1.75 +/- 0.44	1.66 +/- 0.42	1.49 +/- 0.48	<0.0001	31.28 +/- 6.33	38.00 +/- 9.17	<0.0001

mm: millimeters; mm^2^: millimeters square; DIPJ: distal interphalangeal joint; MP: middle phalanx; DP: distal phalanx; Dors: mean dorsal values; Centr: mean central values; Palm: mean palmar values. P-values represent the probability of a significant effect of weight-bearing condition on the measured cartilage thickness and area.

In the frontal plane, the cartilage of the middle phalanx was significantly thicker than that of the distal phalanx in the weight-bearing acquisitions (p = 0.003), while it was thinner in the non-weight-bearing acquisitions (p < 0.0001). In the sagittal plane, a significant difference was observed for the weight-bearing acquisitions (p = 0.0134) only, where the mean thickness of the middle phalanx was greater than that of the distal phalanx, similar to the frontal measurements. A significant interaction was observed between the phalanx and the condition of acquisition in frontal (p < 0.0001) and sagittal (p = 0.001) thickness measurements, as well as in the frontal (p = 0.0015) and sagittal (p = 0.0002) area measurements.

Cartilage thickness significantly differed between the medial, lateral and sagittal aspects of the joint, in both sagittal and frontal planes, both for individual cartilage layers (p = 0.001 on frontal images; p < 0.0001 on sagittal images except for area measurements where p = 0.0195) and DIPJ space measurements (p < 0.0001). On frontal images, cartilage was thinner medially in 7/10 feet (all feet except horse 4 and right front foot of horse 2) during weight-bearing acquisitions, and in 5/10 feet during non-weight-bearing acquisitions. On sagittal images, cartilage was thinner medially in both conditions in all feet except in non-weight-bearing images of the right front foot of horse 2 and the left front foot of horse 3. A significant interaction was found between the location (medial vs lateral) and the condition of acquisition on the frontal (p = 0.0112) as well as sagittal DIPJ (p = 0.0397) thickness measurements, with a larger mediolateral difference observed on weight-bearing compared to non-weight-bearing measurements of the DIPJ thickness in the majority of cases (28/40 measurements in 5/10 feet on frontal images, 6/9 feet on sagittal images).

In sagittal images, the articular cartilage was thinner on the dorsal aspect in a large majority of cases (49/54 measurements in 5/9 feet), on both weight-bearing and non-weight-bearing acquisitions (p < 0.0001). Cartilage was significantly thicker at the palmar and central aspects of the joint on the non-weight-bearing compared with the weight-bearing acquisitions (p < 0.0001), while no significant difference was observed between weight-bearing and non-weight-bearing dorsal individual and combined DIPJ measurements (p = 1).

Results from the blinded reading are summarized in Table 4. Detailed results are available in the S3 Table. A significant difference was observed between individual readers (p < 0.001) as well as between groups (p < 0.001), with better results obtained from the experienced group. No significant difference was observed among the results obtained from individual readers within the same group, except for the students group (p = 0.0287).

**Table 4:** Results obtained during identification of weight-bearing versus non-weight-bearing frontal tridimensional high resolution T1-weighted magnetic resonance images of the distal interphalangeal joint by 3 groups of blinded readers.

	Reader	Images Correctly Identified (%)	Mean % Obtained by Group
**GROUP 1^a^**	1	38/40 (95)	95
	2	38/40 (95)	
	3	38/40 (95)	
**GROUP 2^b^**	4	32/40 (80)	80
	5	32/40 (80)	
	6	32/40 (80)	
**GROUP 3^c^**	7	26/40 (65)	75
	8	36/40 (90)	
	9	28/40 (70)	

^a^ Experienced readers: equine surgeons and large animal imaging residents

^b^ Readers with little experience in equine imaging: small animal imaging residents

^c^ Readers without experience in equine imaging: veterinary students

### Postmortem gross examination

On gross postmortem examination, 4 feet had 1 to 2 focal partial thickness erosions at the palmar aspect of the articular cartilage of the medial condyle of the middle phalanx. One of the feet had a concomitant sagittal fibrillation of the articular cartilage of the distal phalanx. No deeper lesion was observed on any foot.

## Discussion

Difference in cartilage thickness due to load has been demonstrated in human studies [19–21, 25, 26]. It explains the findings of this study where a significantly thinner articular cartilage was observed in the weight-bearing limb compared to the same non-weight-bearing limb. A difference in articular cartilage thickness has been observed in the equine carpus, between antebrachiocarpal and middle carpal joints, [32, 33] but no report discusses in detail different thickness of two opposing articular cartilage surfaces in horses. Differences in biochemical composition and thickness may lead to different cartilage behaviors under load. [33–37] In *ex-vivo* studies of the human femorotibial joint, tibial cartilage deforms and strains markedly more in both compression and shear, than femoral cartilage as a result of femoral cartilage being stiffer. [38] This greater deformation in human tibial cartilage as compared to that of the femoral condyle is also observed with MR imaging following impact loading. [39] The difference between the cartilage thicknesses of the middle and distal phalanges may have similar causes and be related to strain direction and magnitudes varying with joint location and tissue depth, and to difference in biomechanical properties of the cartilage. [40, 41]

Lateromedial foot imbalance is a concern in standing low-field MR imaging, due to limb positioning within the magnet. In fact, limb abduction during standing MR imaging [42] may, in some horses, induce lateromedial asymmetry of DIPJ space, leading to reduction in thickness on the medial aspect as it has been demonstrated on radiographs of normal feet. [43] This joint space asymmetry was also observed in the majority of the feet of the present study, and cartilage margins could not always be outlined on the medial aspect of the joint. This is related to the morphology of the magnet in relation to the height and width of the horse's breast: the smaller or the narrower chested the horse, the more severe will be the abduction and therefore the secondary medial DIPJ compression. Because horse height and breast width were not recorded, correlation between the degree of medial joint space narrowing and size of the horse were not evaluated in the present study, and further studies are needed to investigate this effect.

Only very focal partial thickness erosions and fibrillation were seen at gross post-mortem examination, which were likely too small to be detected with MR imaging. [18] On low-field systems, although T2-weighted fast spin echo sequences demonstrated some utility in detection of metacarpophalangeal cartilage lesions, [8] T1-weighted gradient echo images have been demonstrated to be more accurate for cartilage visualization and delineation [5, 7, 15] as well as for cartilage lesion detection in the DIPJ. [5] Gradient echo sequences are sensitive to chemical shift artefacts, which may lead to false negative results in case of lesions at the osteochondral interface, [7, 8, 44] creating a false appearance of an intact surface adjacent to synovial fluid. However, this artefact is proportional to field strength [45] and its impact on our low-field MR measurements was therefore considered negligible. Therefore, in absence of deep gross postmortem lesions, the results support the hypothesis that asymmetrical narrowing of the DIPJ space during standing MR imaging does not necessarily correspond to pathological cartilage thinning, but is likely the result of asymmetrical loading related to positioning. Future work would be necessary to evaluate the effect of positioning on thinning of articular cartilage when weight-bearing.

A limitation of the present study is the small values of the measurements which were used to test statistical significance despite interpolation, limited spatial resolution and partial volume averaging. Given the low field strength and need for short acquisition times on standing magnets, spatial resolution becomes an issue for evaluating thin structures such as articular cartilage, [18, 30, 44, 45] and partial volume averaging is a major limitation for an accurate cartilage assessment. [5, 8, 12, 46] Furthermore, as the measurements have been performed on a dedicated image processing program, some level of interpolation may also have reduced the accuracy. However, the use of a high-resolution sequence with a pixel size of 0.33 mm resulted in a low error in thickness measurement, particularly when measuring thicker structures like the DIPJ space. [18, 30] As this study aimed to compare articular cartilage thickness values obtained during weight-bearing and non-weight-bearing acquisitions, with similar conditions of acquisition and measurement, and did not aim to obtain reference thickness values of the articular cartilage, the authors believe that the effects of partial volume averaging and interpolation similarly affect both conditions and thus should not impact the statistical differences observed between weight-bearing and non-weight-bearing conditions. A blinded assessment of the images was conducted in order to support the statistical results with a subjective evaluation of differences in cartilage visualization, delineation and thickness between weight-bearing and non-weight-bearing conditions. As the blinded readers correctly identified a large majority of non- vs weight-bearing images based on those criteria, the results of the subjective assessment are similar to quantitative results. The significant difference in results among assessor groups results from a different level of expertise among the groups, with logically better results obtained from the experienced equine practitioners group. The significant difference in results among individuals within the students group is likely to be due to the low level of expertise.

Beyond partial volume averaging, curvature of articular surfaces of the DIPJ is another source of potential error in measurement, as obliquity of the section plane may lead to thickness overestimation. For this reason, the use of several (3–4) oblique frontal planes is recommended in order to be perpendicular to distinct aspects of the articular cartilage while obtaining the measurements. [5] In our study, the weight-bearing frontal images were acquired as perpendicular as possible to DIPJ articular cartilage and attention was paid during non-weight-bearing acquisitions to keep the plane orientation as close as possible to the weight-bearing plane, but no frontal plane was acquired perpendicular to the dorsal aspect of the distal interphalangeal joint, due to practical reasons. This is a limitation from the study since the potential effect of loading on the articular cartilage of the dorsal aspect of the joint has not been assessed on frontal images. However, measurements have been obtained in the dorsal aspect of the joint on the sagittal images, which partly compensated the absence of measurement in the frontal plane.

Finally, the small number of specimens used for this study is due to obvious ethical reasons making this study possible only when particular conditions occurred (horses being available for *in vivo* imaging and being, after a short period of time, euthanized for reasons unrelated to the study). However, the high statistical significance of the majority of our results let us to consider our results reliable despite the limitation of the low sample size.

## Conclusions

The results of the present study demonstrate that the articular cartilage of the DIPJ thins when loaded compared to post mortem isolated decreased weight-bearing distal limbs and differences exist between cartilage deformation in the middle and distal phalanges. Some cartilage abnormalities easily visualized on non-weight-bearing limbs may therefore be more difficult to identify on standing foot MR imaging when asymmetrical joint space thinning due to positioning is a common feature and DIPJ cartilage is compressed and sheared. Caution should therefore be recommended in interpreting articular cartilage on weight-bearing limbs and in using cartilage thickness values obtained on non-weight-bearing limbs as a reference for standing MR imaging on clinical cases.

## Supporting information

S1 Table Frontal measurements data. (CSV)

S2 Table Sagittal measurements data. (CSV)

S3 Table Results of blinded reading. (CSV)
